# Supplementary material for: Activation of Nrf2/HO-1 signaling pathway exacerbates cholestatic liver injury
Source: Commun Biol. 2024 May 23;7:621. doi: 10.1038/s42003-024-06243-0 (PMC11116386; doi:10.1038/s42003-024-06243-0)
Supplement: Supplementary file 2 — Supplementary Information [file 42003_2024_6243_MOESM2_ESM.pdf]

Activation of Nrf2/HO-1 signaling pathway exacerbates cholestatic liver injury

Yi Wang[1,2,#], Xiaolong Fu[1,2,#], Li Zeng[1,2], Yan Hu[1,2], Rongyang Gao[1,2], Siting Xian[1,2], Songjie Liao[1,2], Jianxiang Huang[1,2], Yonggang Yang[1,2], Jilong Liu[3], Hai Jin[4], James Klaunig[5], Yuanfu Lu[1,2,*] & Shaoyu Zhou[1,2,*]

[1]Key Laboratory of Basic Pharmacology of Ministry of Education and Joint International Research Laboratory of Ethnomedicine of Ministry of Education, Zunyi Medical University, Zunyi, China

[2]School of Pharmacy, Zunyi Medical University, Zunyi, China

[3]Department of Gastroenterology, Digestive Disease Hospital, Affiliated Hospital of Zunyi Medical University, Zunyi, China

[4]Institute of Digestive Diseases of Affiliated Hospital, Affiliated Hospital of Zunyi Medical University, Zunyi, China

[5]Department of Environmental and Occupational Health, School of Public Health, Indiana University, Bloomington, Indiana, USA

[#]These authors contributed equally to this work.

Correspondence should be addressed to:

Dr. Shaoyu Zhou or Dr Yuanfu Lu

E-mail: szhou@zmu.edu.cn or luyuanfu2000@163.com

Supplementary Figure 1

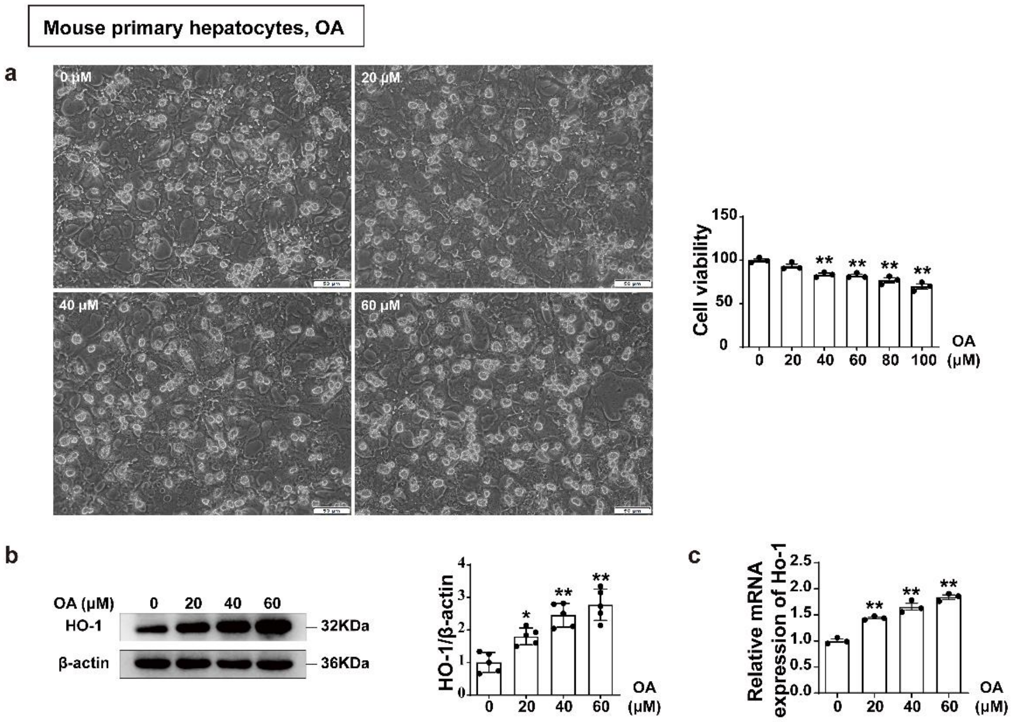

Supplementary Figure 1. Expression of HO-1 protein and mRNA in mouse primary hepatocytes. a Cell morphology and cell viability (n=3). **b** Protein expression level of HO-1 (n=5). **c** mRNA expression level of *Ho*-1 (n=3). One-way ANOVA (**b, c**) with Tukey's post hoc test or nonparametric tests (**a**) was used. The data is shown as the Mean ± SEM. $^{*}p < 0.05$, $^{**}p < 0.01$, significant difference compared to the control group. HO-1, heme oxygenase-1; OA, oleanolic acid.

Supplementary Figure 2

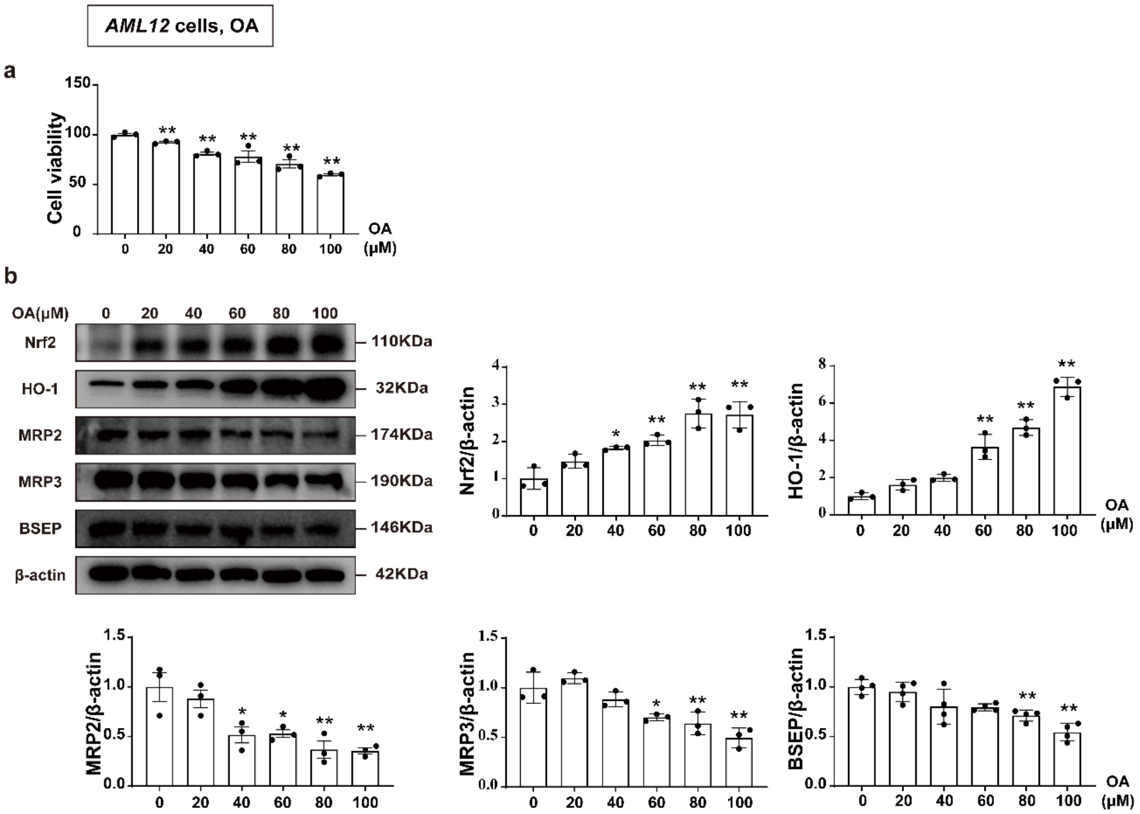

Supplementary Figure 2. Expression of Nrf2, HO-1 and hepatobiliary transporters in *AML12* cells. a Cell viability (n=3). **b** Protein level of Nrf2, HO-1, MRP2, MRP3 and BSEP (n=3-4). One-way ANOVA (**a, b**) with Tukey's post hoc test or Games-Howell analysis or nonparametric statistical test was used. The data are shown as the Mean ± SEM. $^{*}p < 0.05$, $^{**}p < 0.01$, significant difference compared to the control group. BSEP, bile salt export pump; HO-1, heme oxygenase-1; MRP, multidrug resistance protein; Nrf2, nuclear factor erythroid 2-related factor 2; OA, oleanolic acid.

Supplementary Figure 3

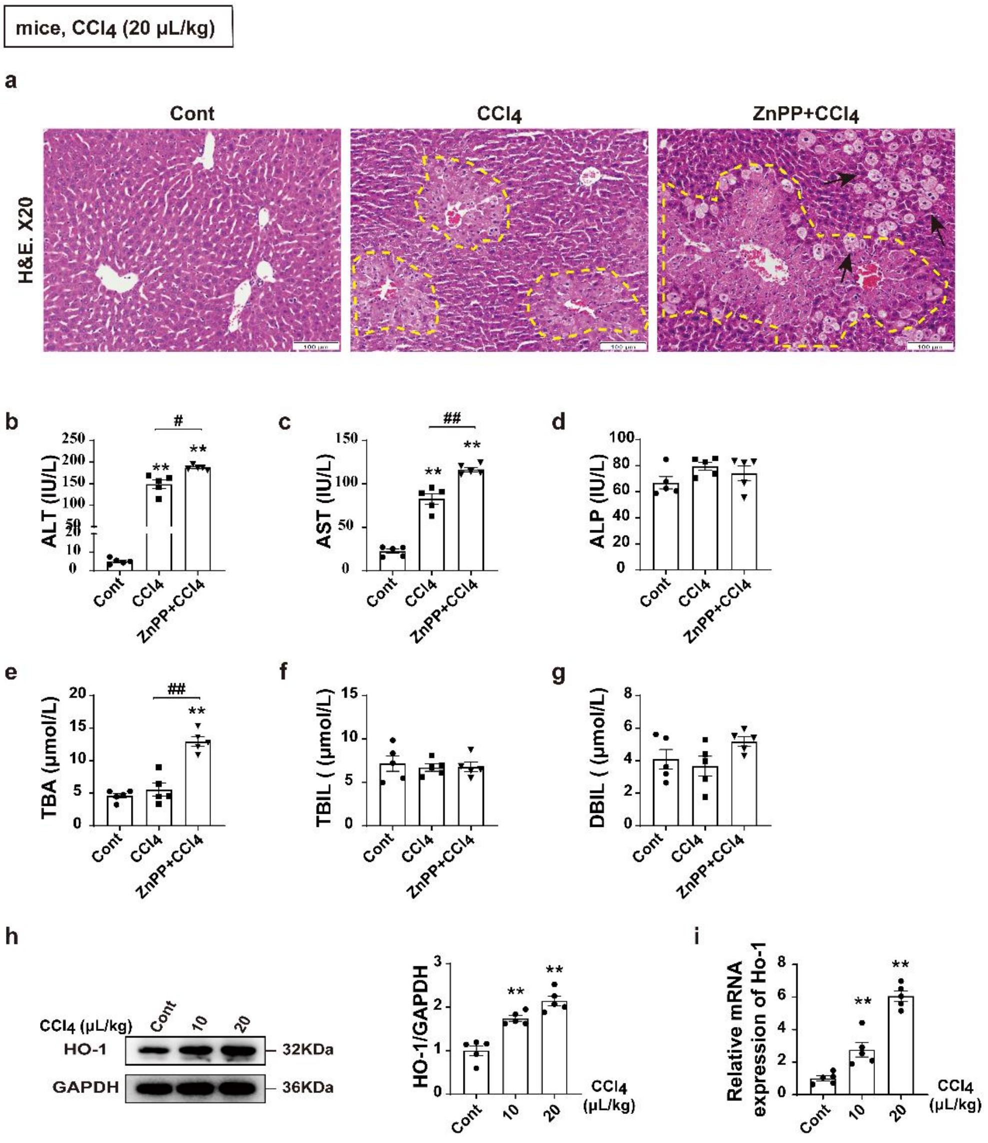

Supplementary Figure 3. HO-1 expression in mice with CCl_4-induced cholestatic liver injury. a Representative images of H&E staining (scale bar, 100 μm) (n=5). Ballooning degeneration (black arrows) and hepatocyte necrosis (yellow dashed circles). **b-g** Serum contents of ALT, AST, ALP, TBA, TBIL and DBIL (n=5). **h, i** Protein and mRNA expression level of HO-1 (n=5). Student's t test and nonparametric tests were used. The data are shown as the Mean ± SEM. $^{*}p < 0.05$, $^{**}p < 0.01$, significant difference compared to the control group; $^{\#}p < 0.05$, $^{\#\#}p < 0.01$, significant difference between the CCl_4 alone group and the ZnPP-combined group. ALT, alanine aminotransferase; AST, aspartate aminotransferase; DBIL, direct

bilirubin; H&E, hematoxylin and eosin; HO-1, heme oxygenase-1; TBA, total bile acid; TBIL, total bilirubin.

Supplementary Figure 4

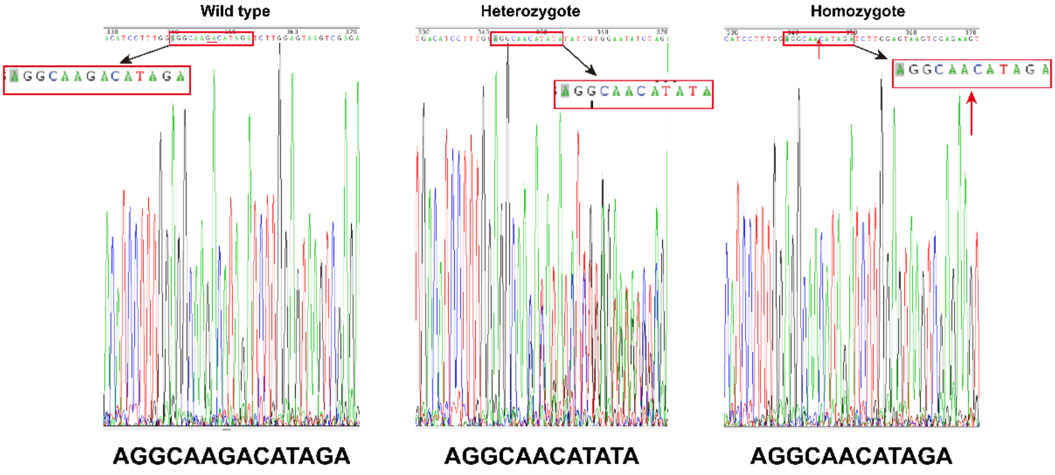

Supplementary Figure 4. Gene identification of Nrf2 knockout mice.

Supplementary Figure 5

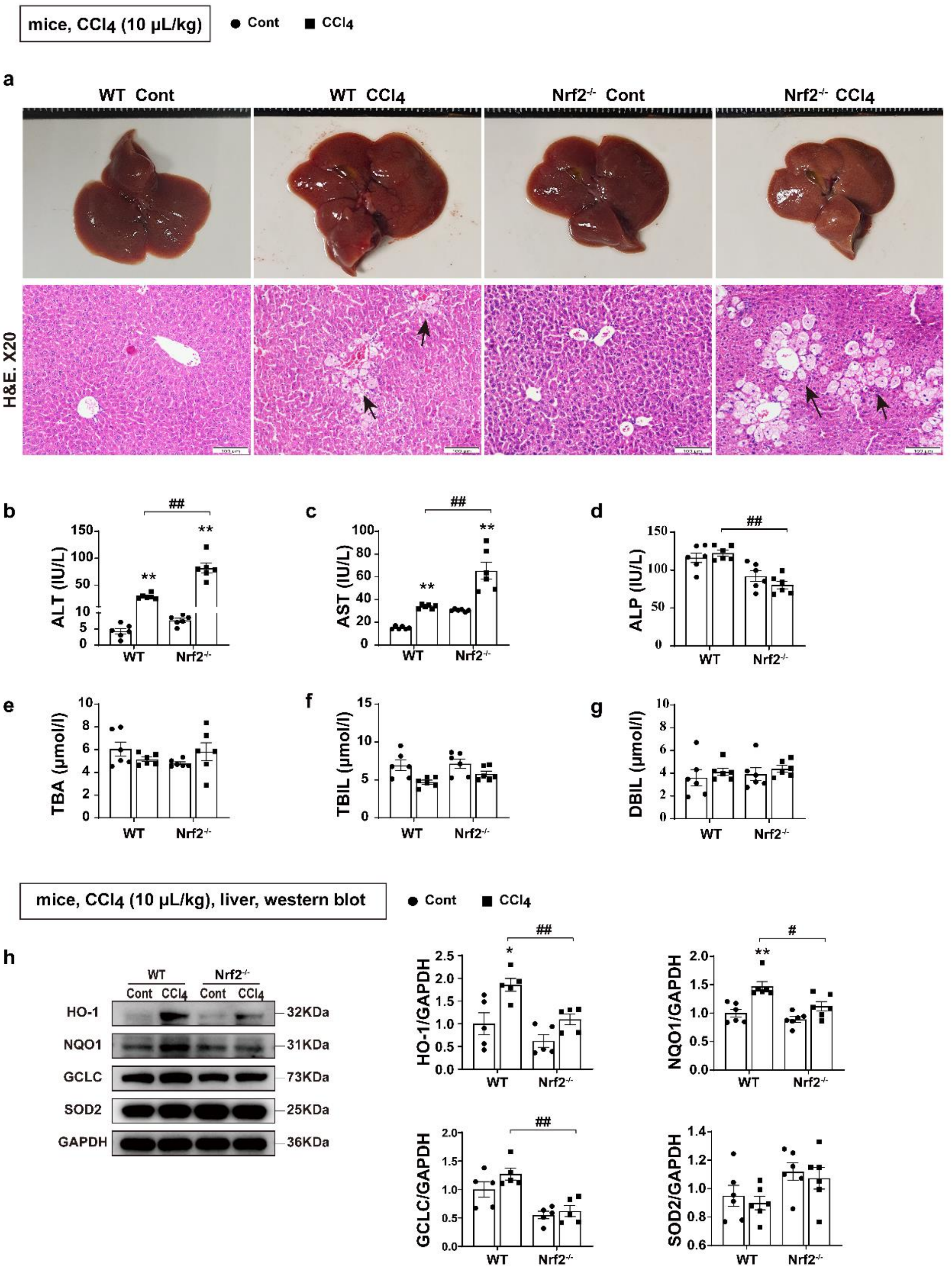

Supplementary Figure 5. Expression of Nrf2/HO-1 mediated antioxidant proteins in liver mice with CCl_4-induced liver injury. a Representative images of liver gross examination and H&E staining (scale bar, 100 μm) (n=6). Ballooning

degeneration (black arrows). **b-h** Serum contents of ALT, AST, ALP, TBA, TBIL and DBIL (n=6). **i** Protein expression level of HO-1, NQO1, GCLC and SOD2 in liver tissues (n=5-6). Student's t test (**b-i**) and nonparametric tests (**i**: NQO1/GAPDH) were used. The data are shown as the Mean ± SEM. $^{*}p < 0.05$, $^{**}p < 0.01$, significant difference compared to the control group; $^{\#}p < 0.05$, $^{\#\#}p < 0.01$, significant difference between the WT CCl_4 group and the *Nrf2*$^{-/-}$ CCl_4 group. ALP, alkaline phosphatase; ALT, alanine aminotransferase; AST, aspartate aminotransferase; CCl_4, carbon tetrachloride; DBIL, direct bilirubin; GCLC, glutamate-cysteine ligase catalytic subunit; H&E, hematoxylin and eosin; HO-1, heme oxygenase-1; NQO1, NAD(P)H-quinone oxidoreductase 1; SOD2, superoxide dismutase 2; TBA, total bile acid; TBIL, total bilirubin.

Supplementary Figure 6

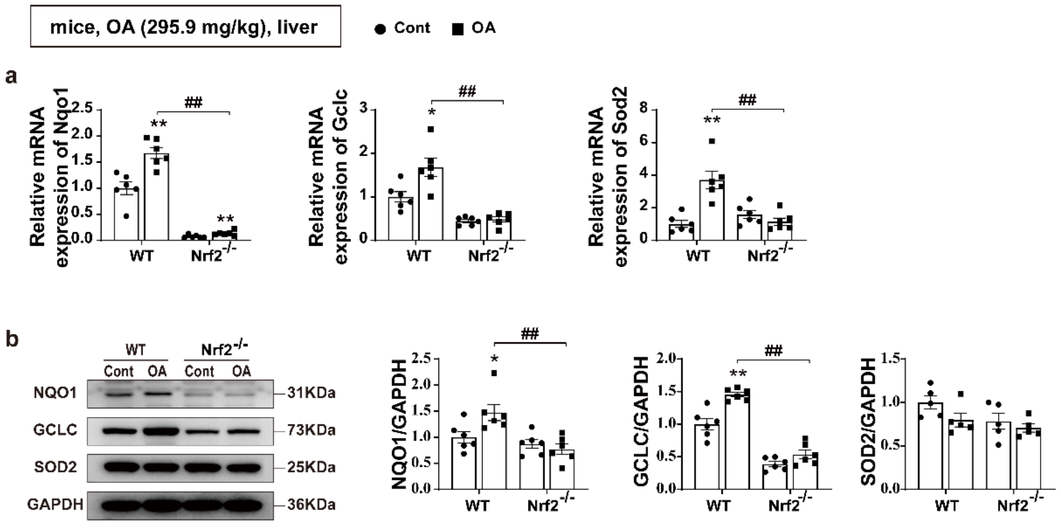

Supplementary Figure 6. Nrf2/HO-1 mediated expression of antioxidant proteins and genes in mice with OA-induced cholestatic liver injury. a The mRNA expression level of *Nqo1*, *Gclc* and *Sod2* (n=6). **b** Protein expression level of NQO1, GCLC and SOD2 (n=5-6). Student's t test (**a, b, c**) was used. The data are shown as the Mean ± SEM. $^{*}p < 0.05$, $^{**}p < 0.01$, significant difference compared to the control group; $^{\#}p < 0.05$, $^{\#\#}p < 0.01$, significant difference between the WT OA group and the *Nrf2*$^{-/-}$ OA group. GCLC, glutamate-cysteine ligase catalytic subunit; NQO1, NAD(P)H-quinone oxidoreductase 1; OA, oleanolic acid; SOD2, superoxide dismutase 2.

Supplementary Figure 7

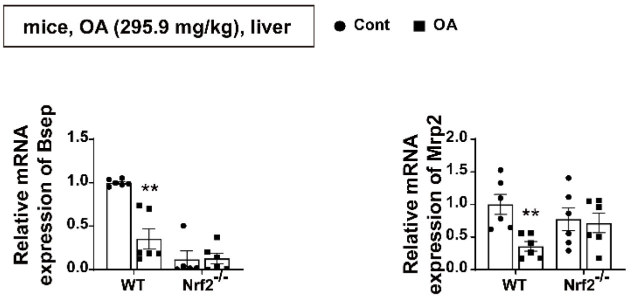

Supplementary Figure 7. Nrf2/HO-1 mediated mRNA expression of *Bsep* and *Mrp2* in the liver of mice with OA-induced cholestatic liver injury (n=5-6). Student's t test was used. The data are shown as the Mean ± SEM. $^{*}p$ < 0.05, $^{**}p$ < 0.01, significant difference compared to the control group. BSEP, bile salt export pump; MRP, multidrug resistance protein.

Supplementary Figure 8

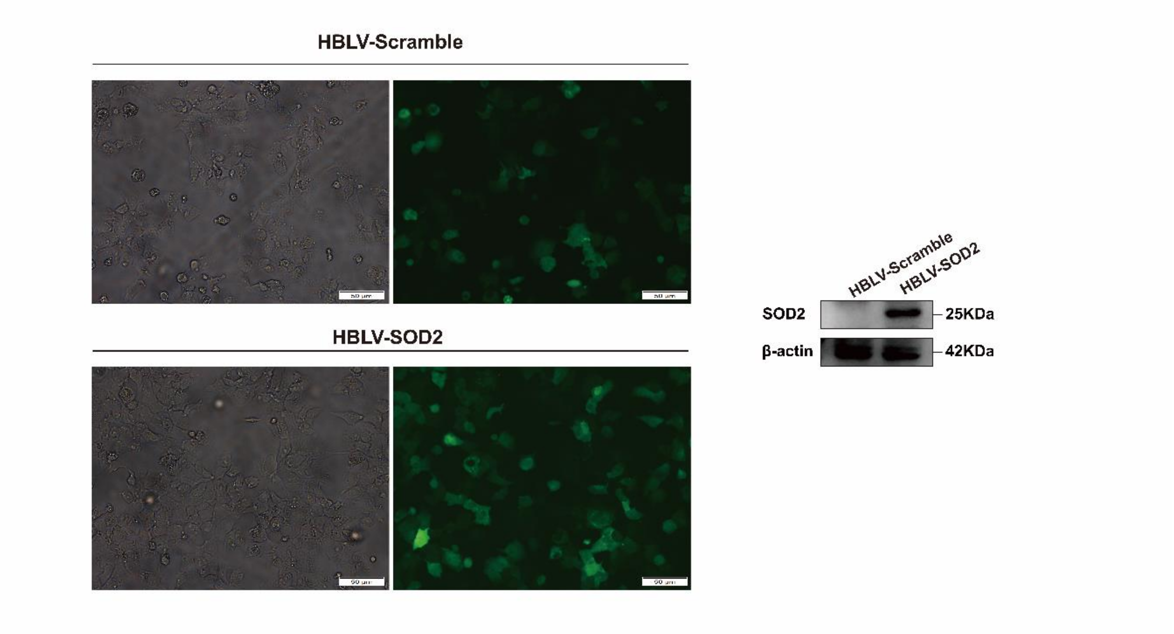

Supplementary Figure 8. The transfection efficiency of LV-SOD2 in *AML12* cells. Representative green fluorescence and western blotting were conducted to assess the transfection efficiency of LV-SOD2 in *AML12* cells. Green fluorescence indicates the expression of SOD2. SOD2, superoxide dismutase 2.

Supplementary Figure 9

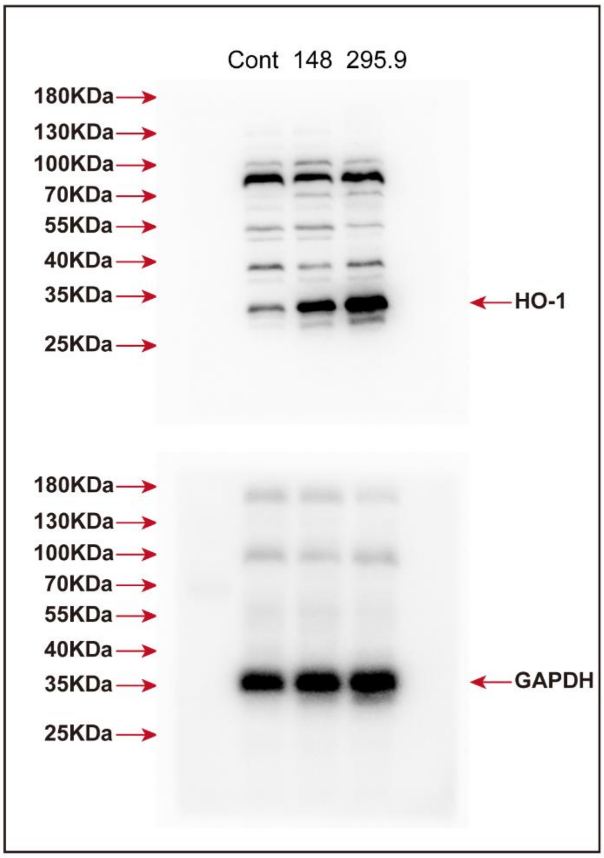

a) Uncropped gel images of Figure 1i

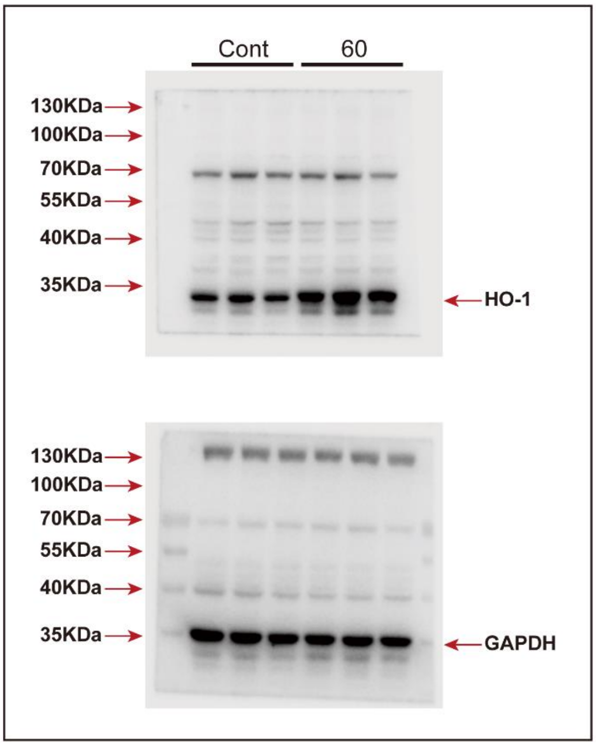

b) Uncropped gel images of Figure 1l

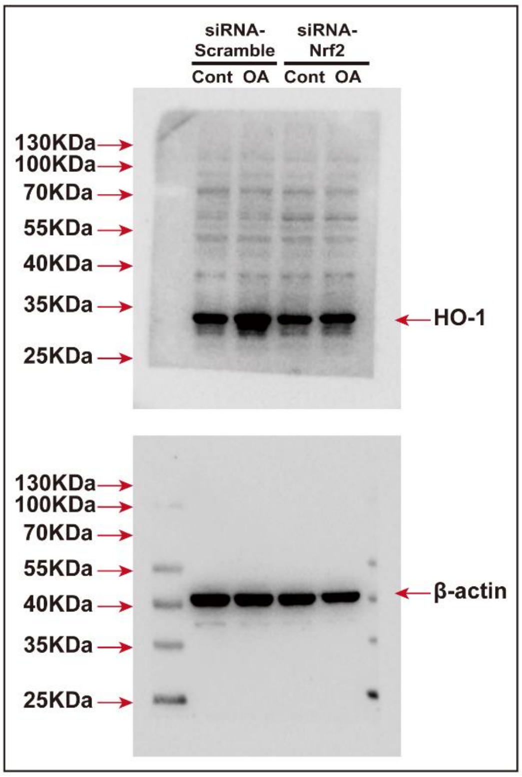

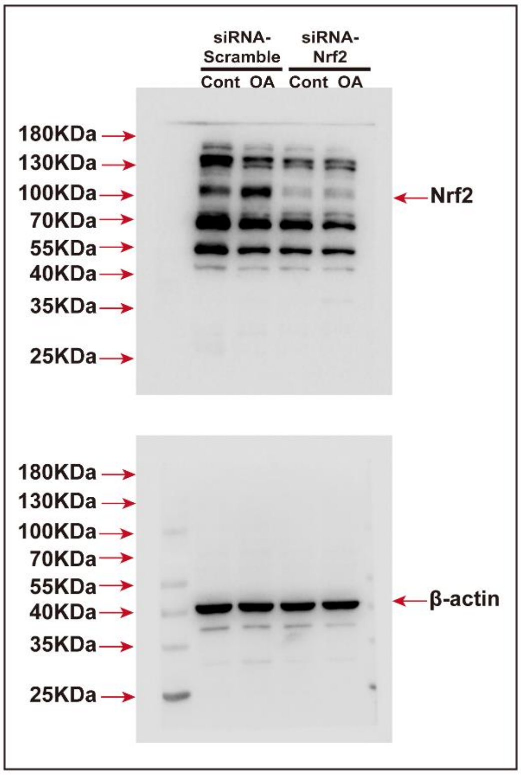

c) Uncropped gel images of Figure 2b

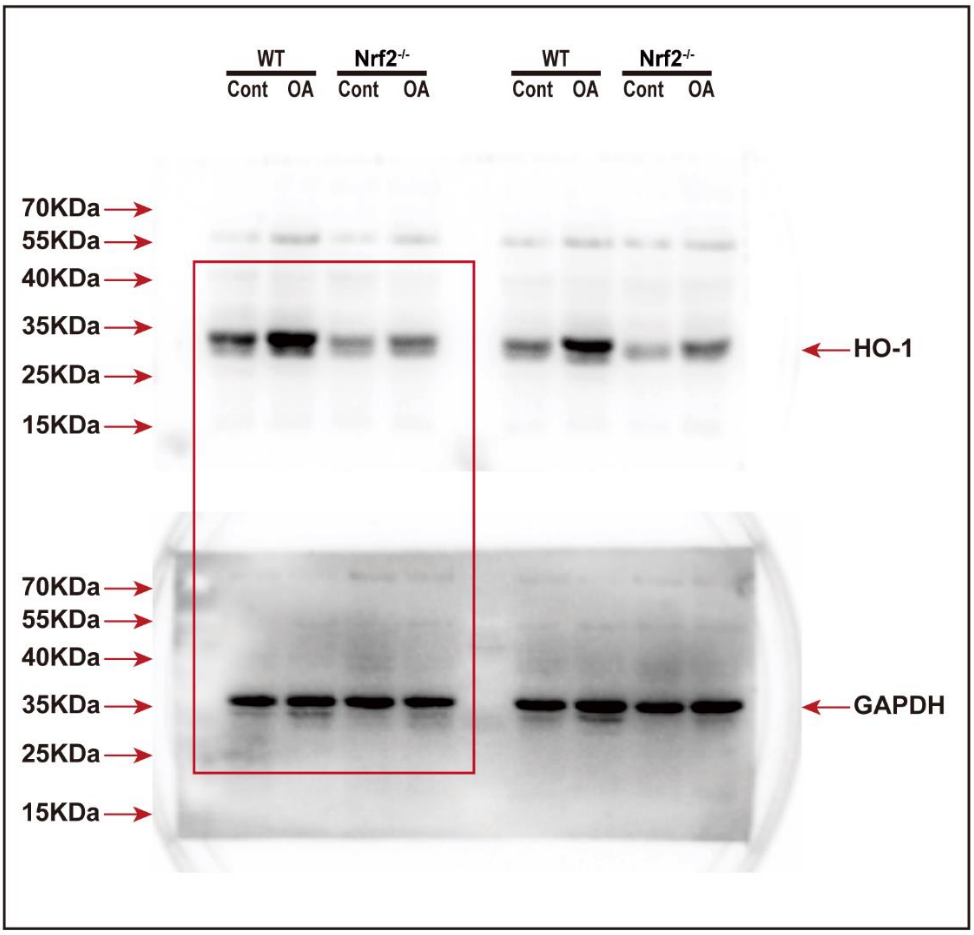

d) Uncropped gel images of Figure 2m

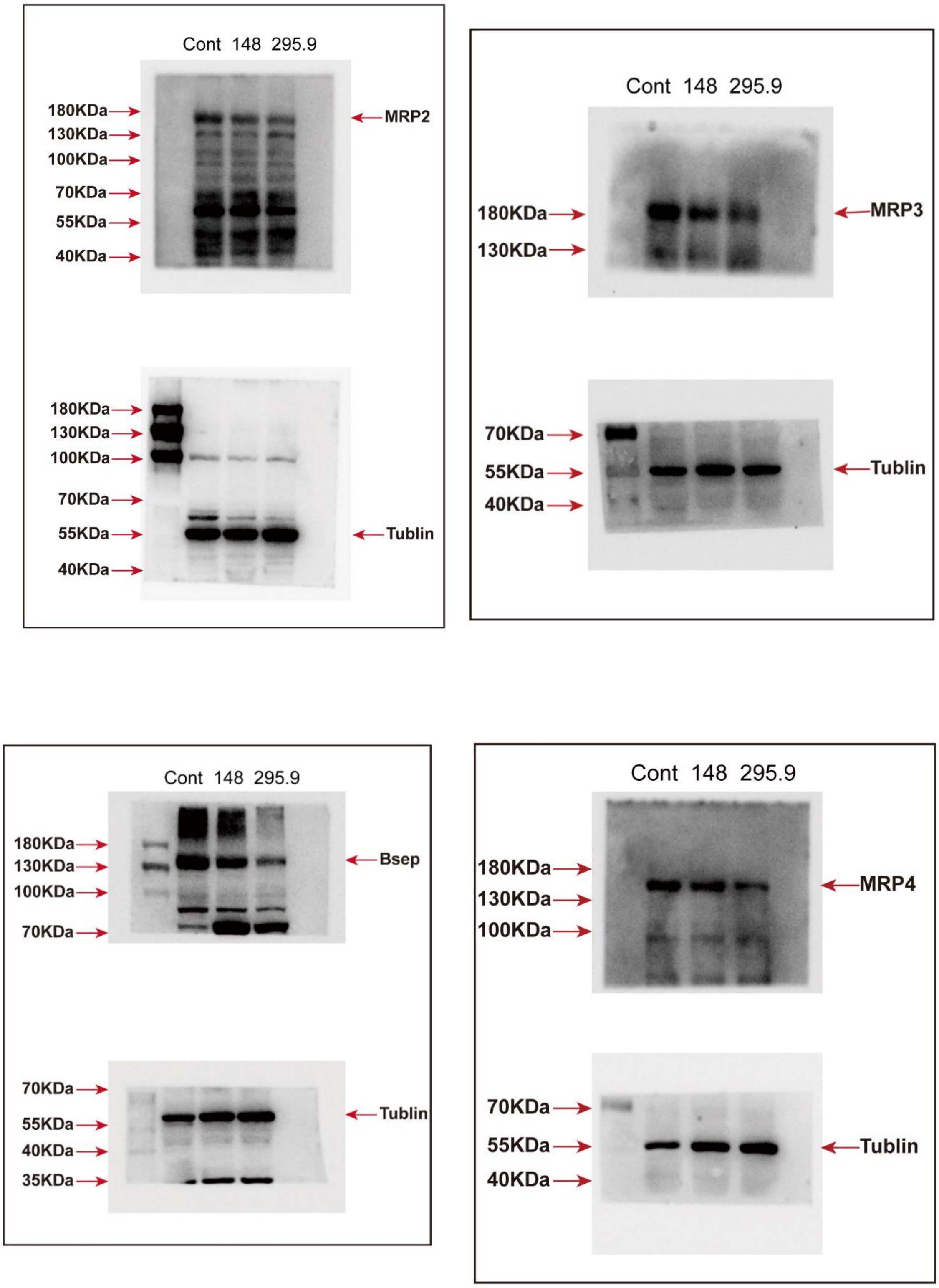

e) Uncropped gel images of Figure 4a

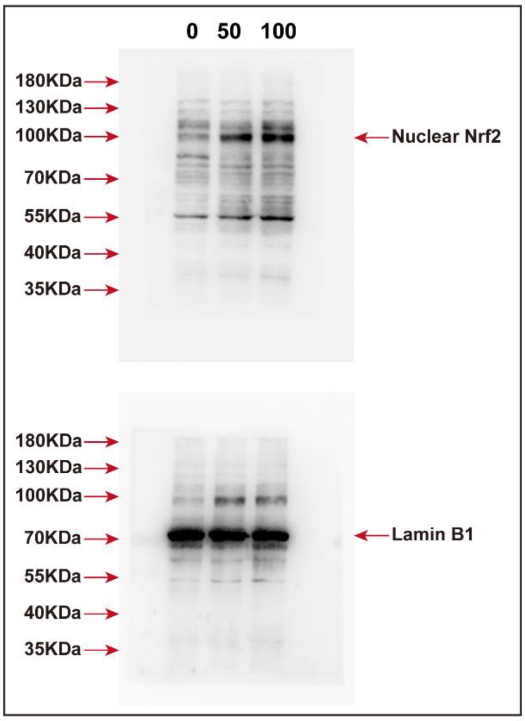

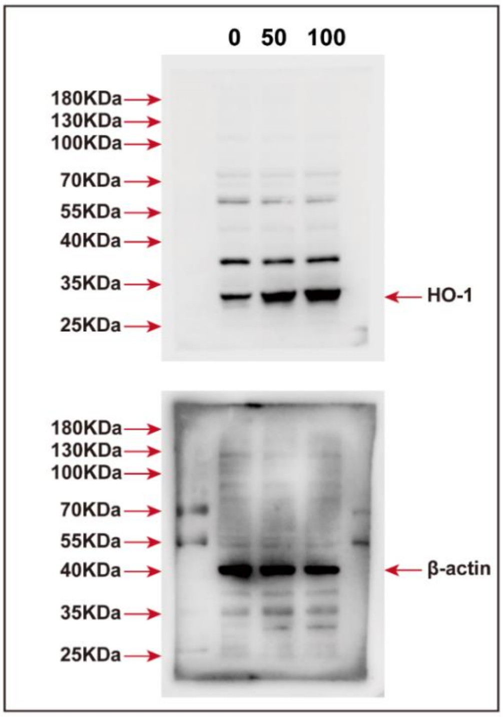

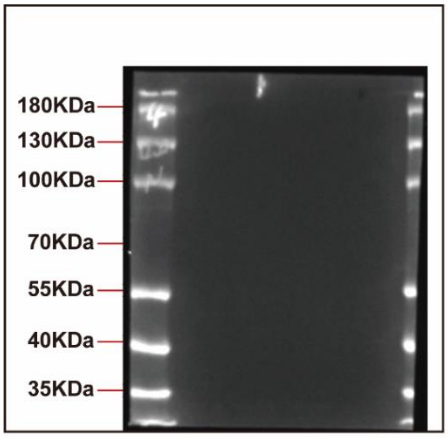

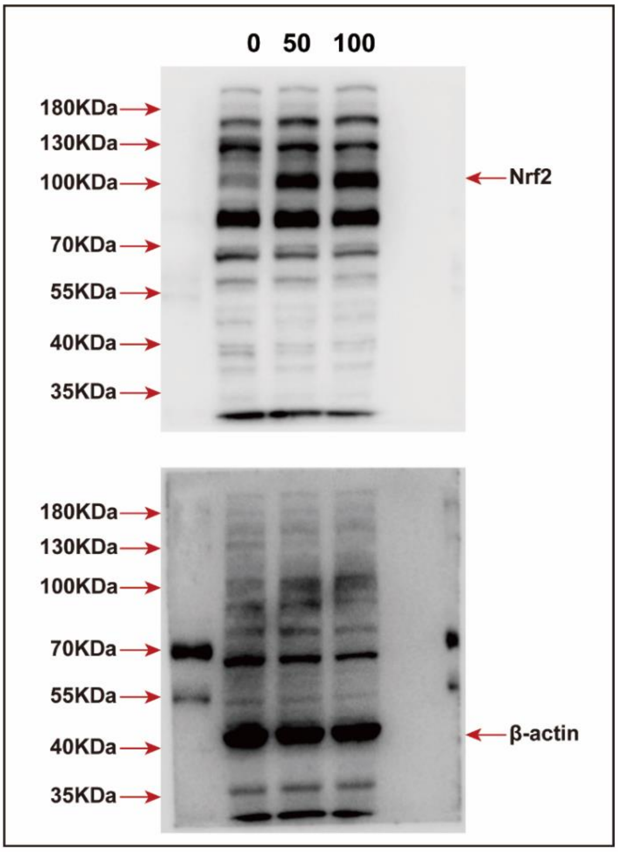

f) Uncropped gel images of Figure 5g

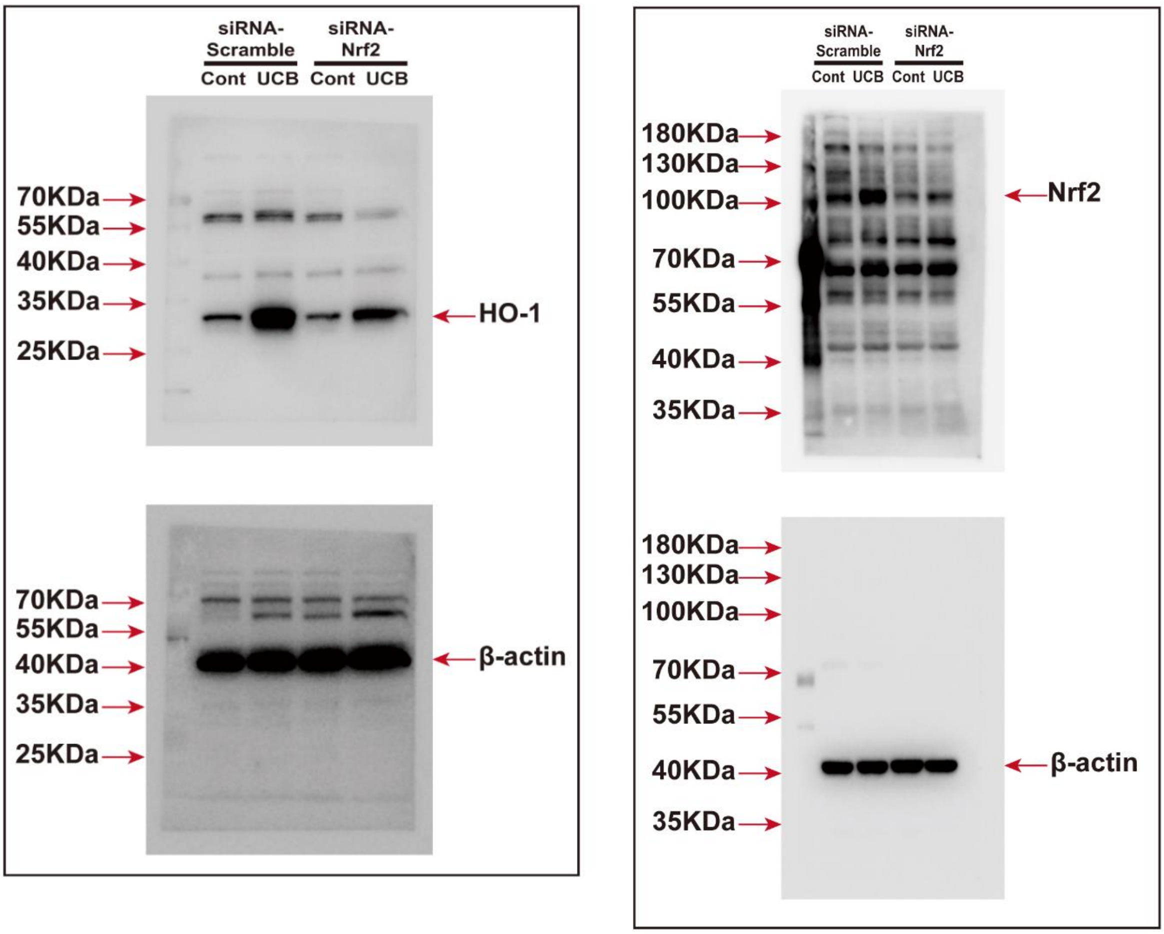

g) Uncropped gel images of Figure 8a

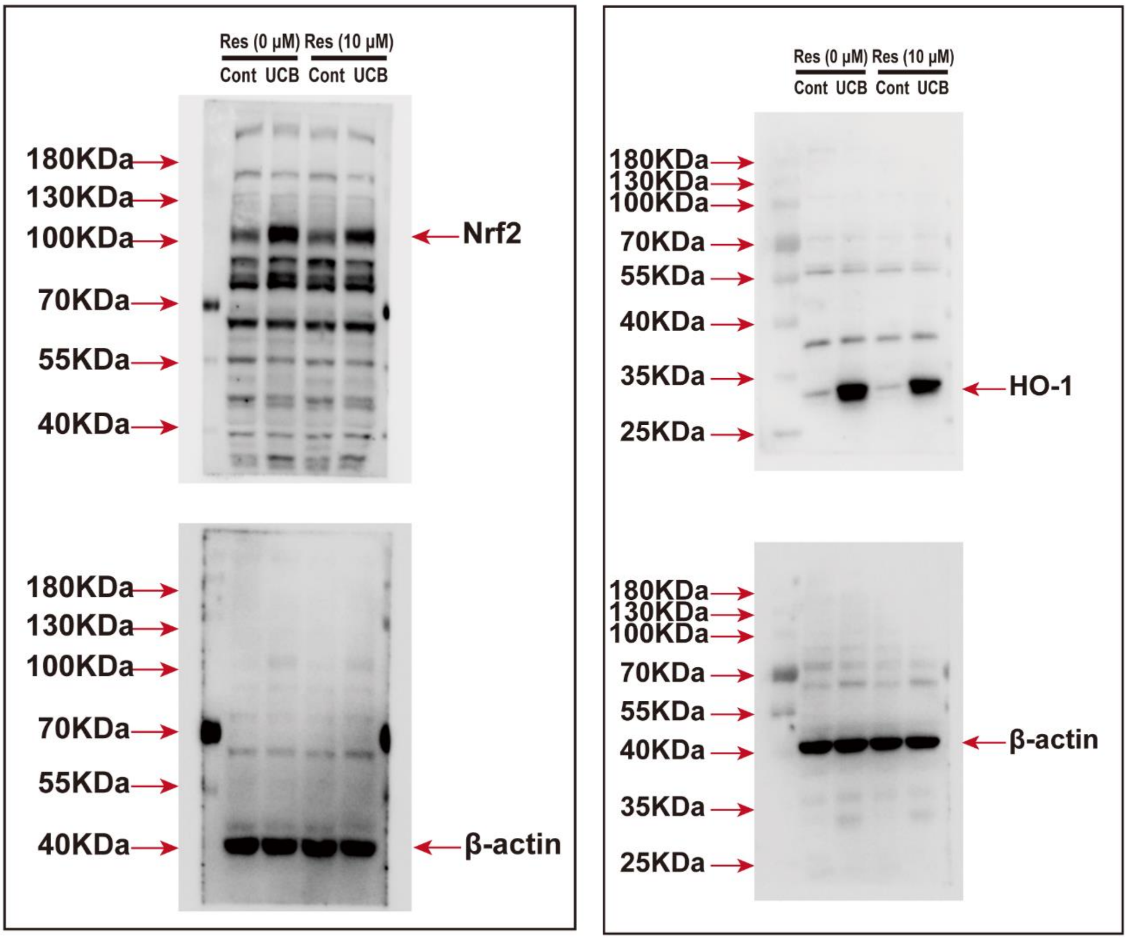

h) Uncropped gel images of Figure 8c

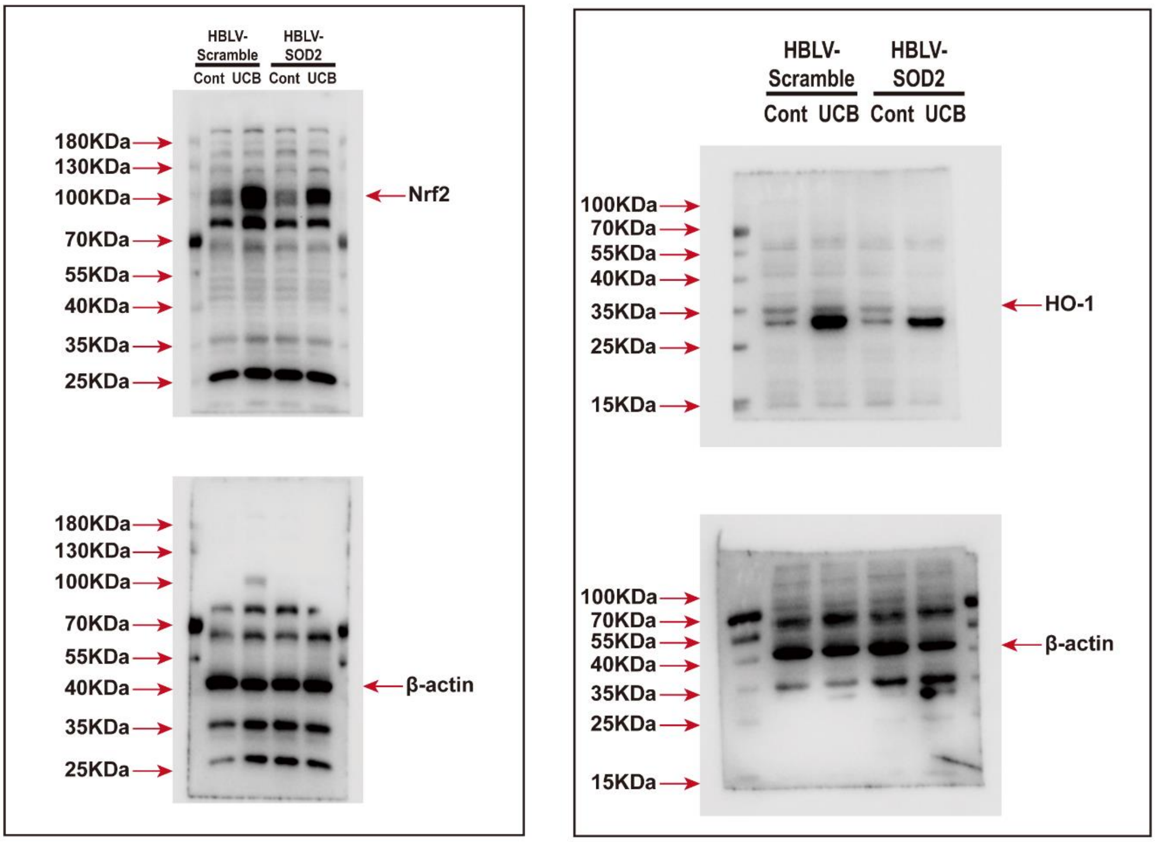

i) Uncropped gel images of Figure 8e

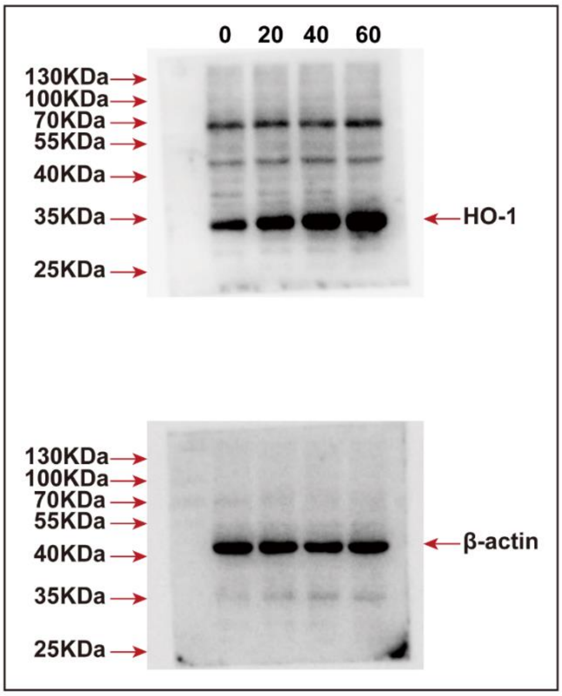

j) Uncropped gel images of Figure S1b

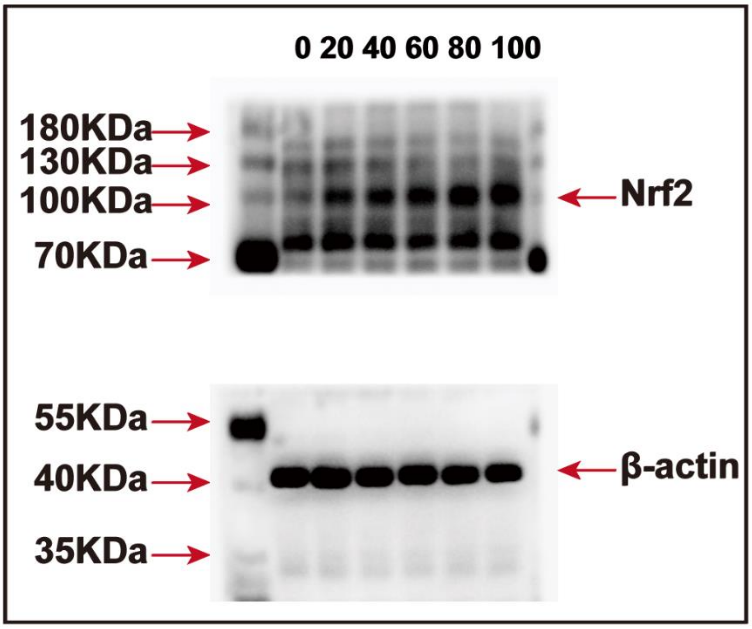
0 20 40 60 80 100
180KDa
130KDa
100KDa
70KDa
Nrf2
55KDa
40KDa
35KDa
β-actin

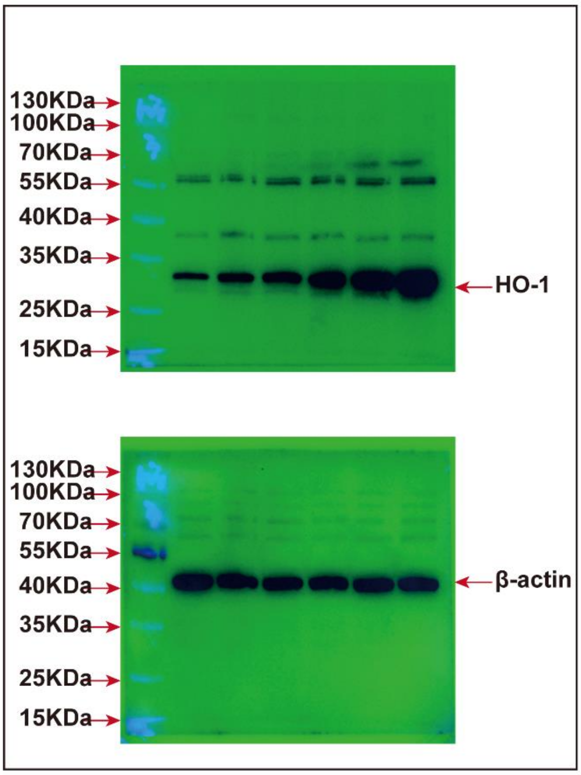
130KDa
100KDa
70KDa
55KDa
40KDa
35KDa
25KDa
15KDa
HO-1
130KDa
100KDa
70KDa
55KDa
40KDa
35KDa
25KDa
15KDa
β-actin

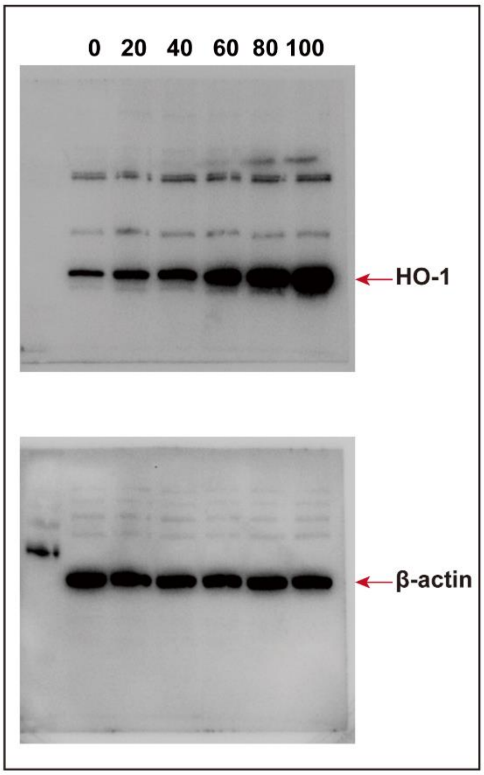
0 20 40 60 80 100
HO-1
β-actin

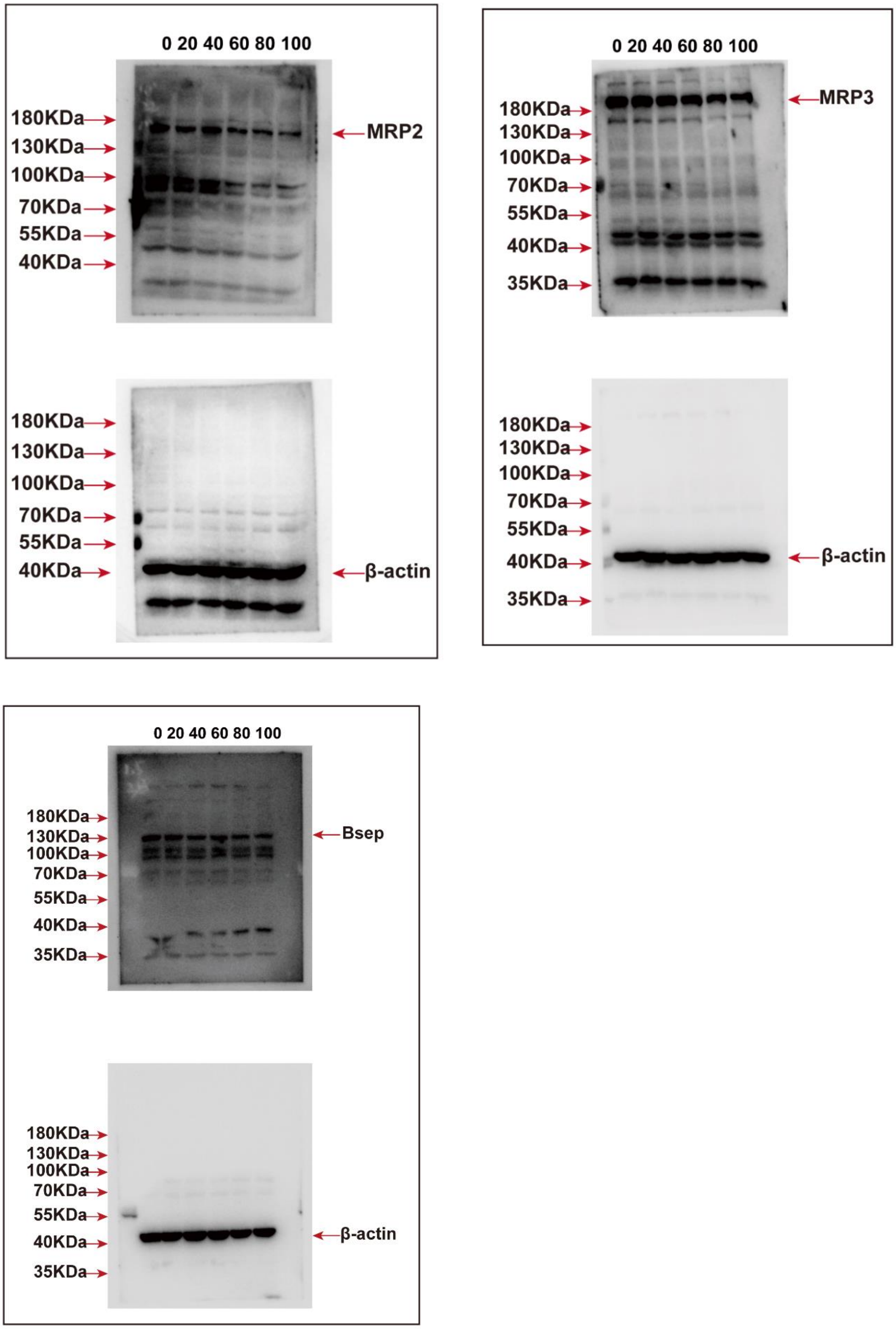

k) Uncropped gel images of Figure S2b

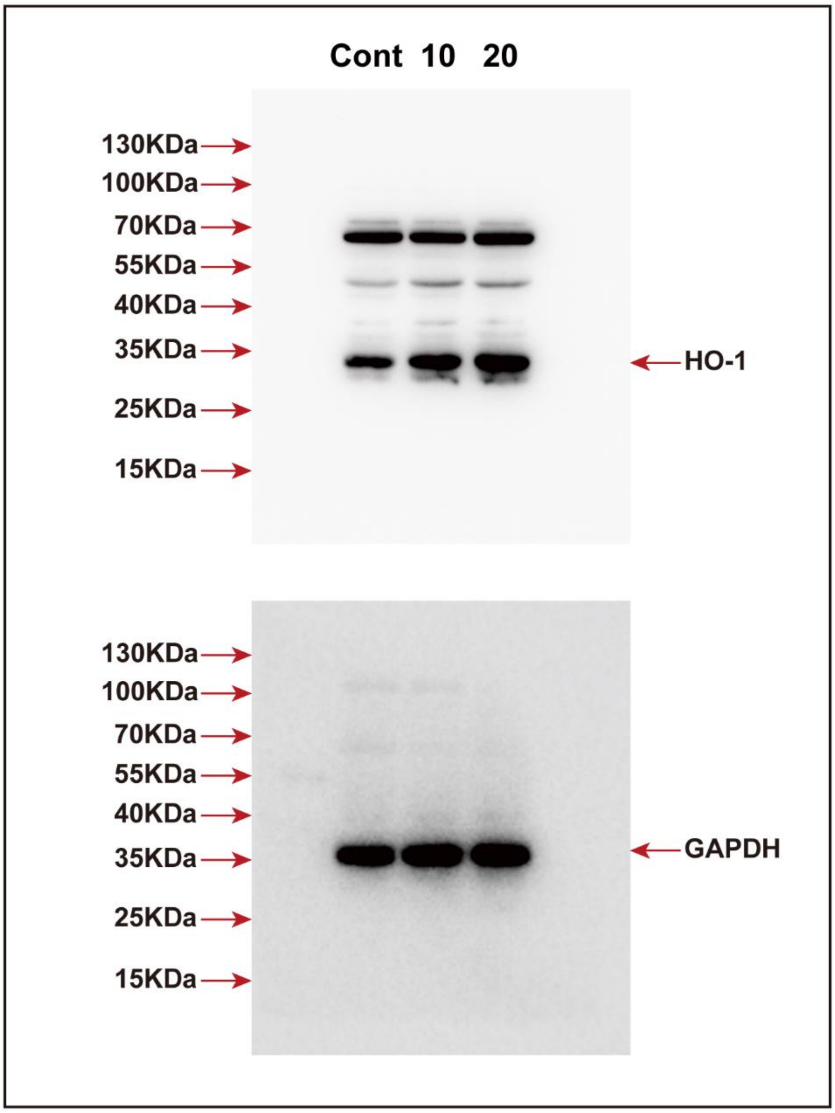

l) Uncropped gel images of Figure S3h

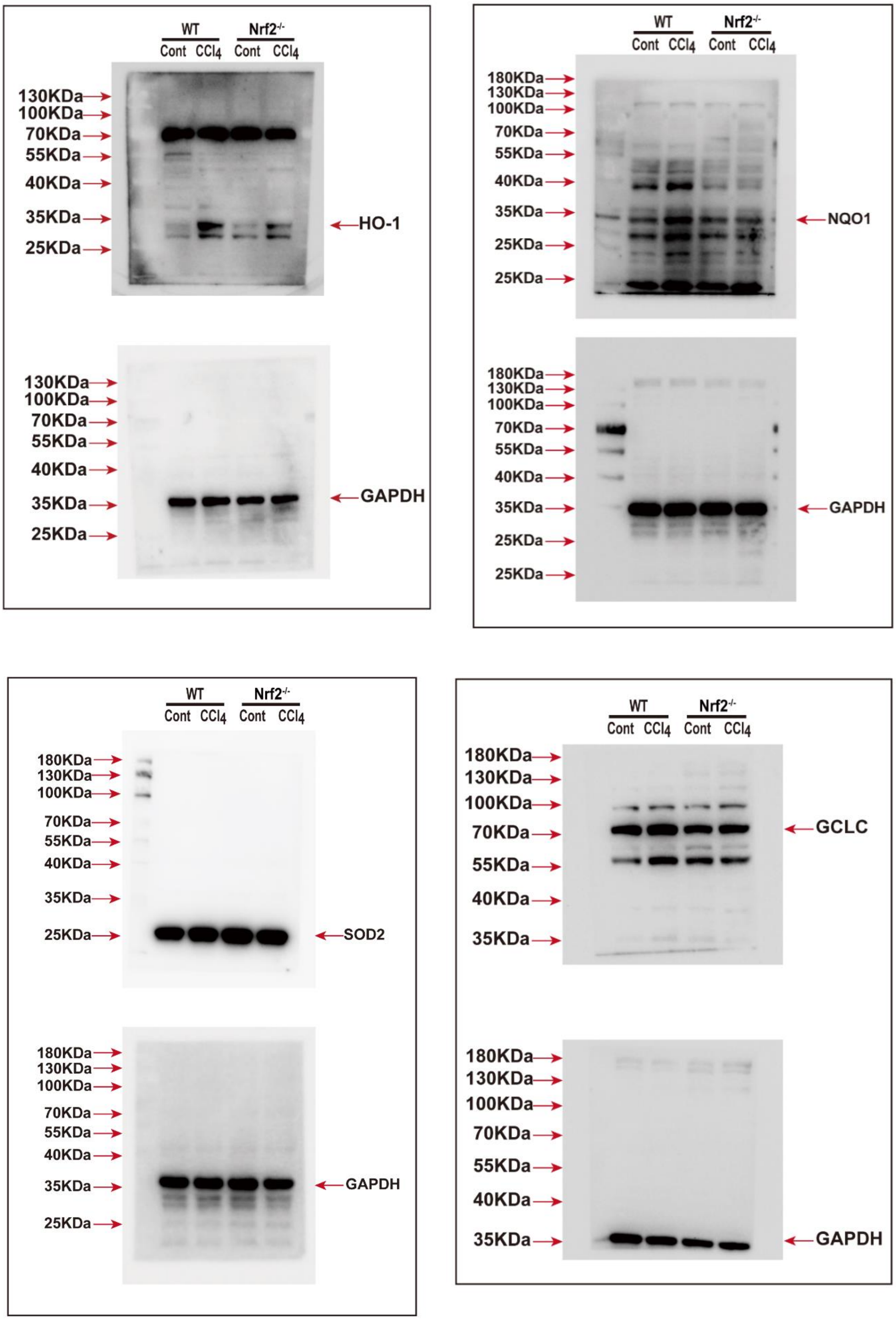

m) Uncropped gel images of Figure S5h

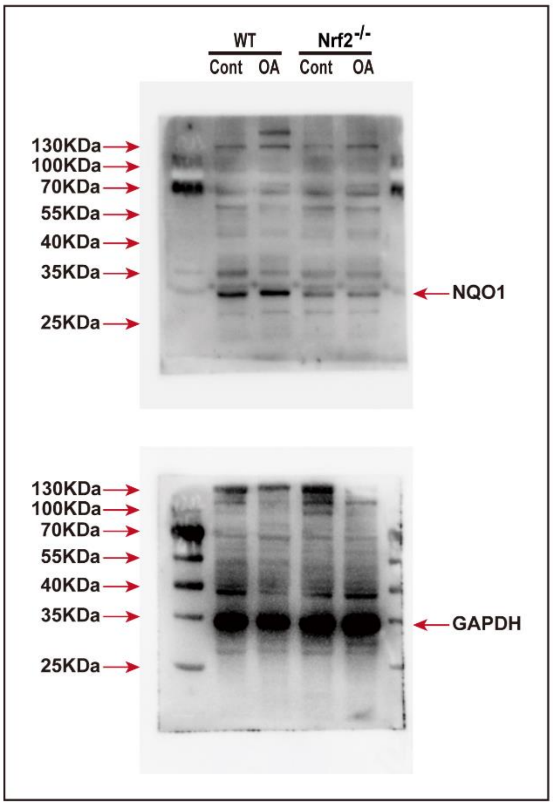

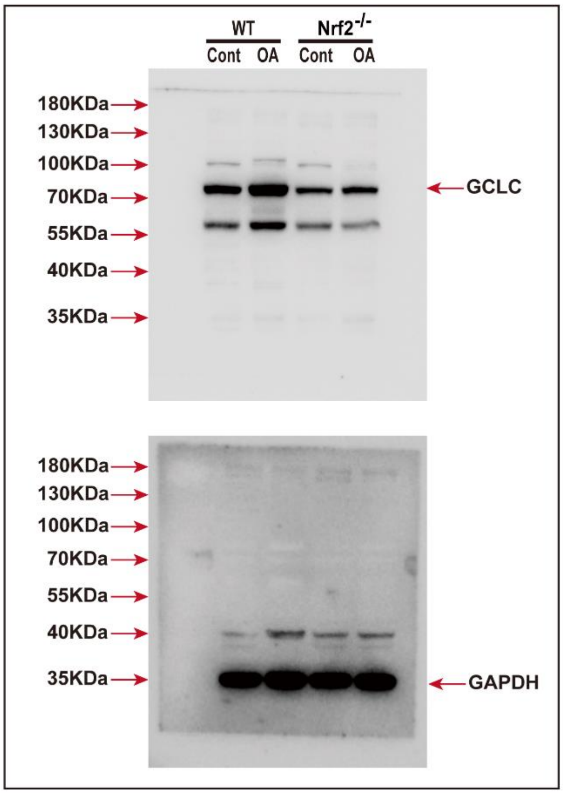

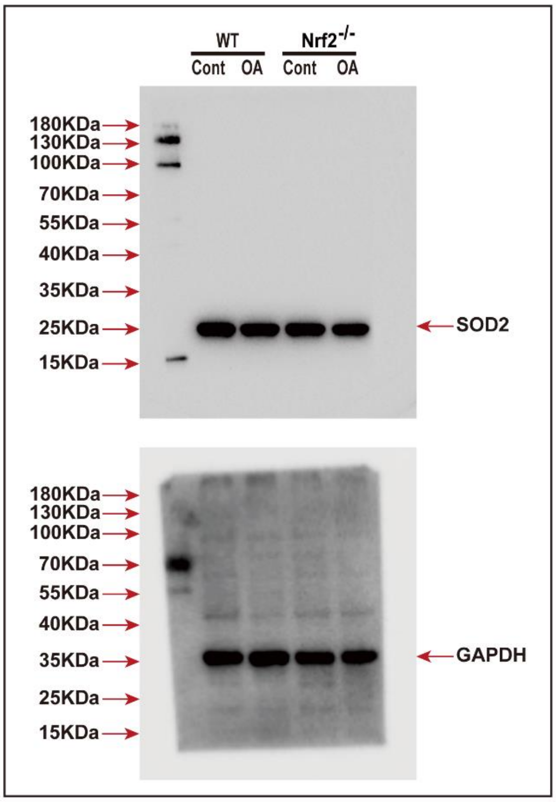

n) Uncropped gel images of Figure S6b

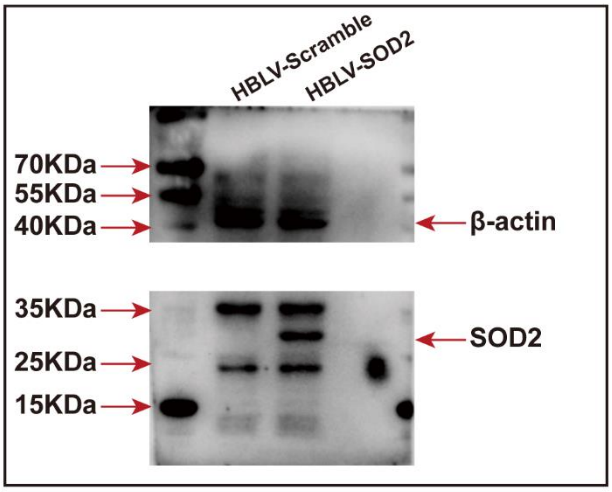

o) Uncropped gel images of Figure S8

Supplementary Table 1. Biochemical measurements in serum of normal individuals (n=27) and patients (n=61).

Indicators	Normal	Patient
ALT	17.56 ± 3.08	61.61 ± 9.88 [a]
AST	22.41 ± 1.55	61.90 ± 5.91 [a]
ALP	68.70 ± 4.72	202 ± 34.07 [a]
GGT	19.48 ± 2.26	201.15 ± 27.12 [a]
CHE	7.42 ± 0.43	4.09 ± 0.26 [a]
TBIL	12.55 ± 1.10	66.54 ± 11.00 [a]
DBIL	3.59 ± 0.74	28.97 ± 5.40 [a]
IBIL	8.96 ± 0.88	37.04 ± 6.08 [a]
TBA	3.53 ± 0.54	66.30 ± 11.67 [a]
TP	69.50 ± 1.16	60.84 ± 1.06 [a]
ALB	45.16 ± 1.57	33.93 ± 0.66 [a]
GLB	25.22 ± 0.60	26.55 ± 1.03
A/G	1.76 ± 0.07	1.77 ± 0.42 [a]
PA	235.89 ± 13.06	110.61 ± 8.36 [a]

Note: Serum samples were obtained from 27 normal individuals and 61 CLI patients. The data are shown as the mean ± SEM. $^{a}p < 0.01$, significant difference between the normal group and the patient group. A/G, albumin/globulin; ALB, albumin; ALP, alkaline phosphatase; ALT, alanine aminotransferase; AST, aspartate aminotransferase; CHE, cholinesterase; DBIL, direct bilirubin; IBIL, indirect bilirubin; GGT, gamma glutamyl trans; GLB, globulin; PA, preprotein; TBA, total bile acid; TBIL, total bilirubin; TP, total protein.

Supplementary Table 2. Sequences of Real-time qPCR primers.

Genes	Forward primers (5'-3')	Reverse primers (5'-3')
Gapdh (human)	ATGACCCCTTCATTGACC	GAAGATGGTGATGGGATTTC
Ho-1 (human)	CCAGGCAGAGAATGCTGAGTTC	AAGACTGGGCTCTCCTTGTTGC
Nrf2 (human)	CACATCCAGTCAGAAACCAGTGG	GGAATGTCTGCGCCAAAAGCTG
Bsep (mouse)	ACAGCACTACAGCTCATTCAGAG	TCCATGCTCAAAGCCAATGATCA
Gapdh (mouse)	TGTGTCCGTCGTGGATCTGA	CCTGCTTCACCACCTTCTTGA
Gclc (mouse)	AACACAGACCCAACCCAGAG	CCGCATCTTCTGGAAATGTT
Ho-1 (mouse)	CACAGATGGCGTCACTTCGTC	GTGAGGACCCACTGGAGGAG
Mrp2 (mouse)	GCACTGTAGGCTCTGGGAAG	CATTTCCAAGTCTGGGAGGA
Nqo1 (mouse)	CAGATCCTGGAAGGATGGAA	TCTGGTTGTCAGCTGGAATG
Sod2 (mouse)	GCCCCCTGAGTTGTTGAATA	AGACAGGCAAGGCTCTACCA

Supplementary Table 3. Information of antibodies for Western blotting.

Antibodies	Source	Catalog number	Antibody concentration
BSEP	Invitrogen	PA5-78690	1:1000
GAPDH	Proteintech	60004–1-Ig	1:5000
GCLC	Proteintech	12601-1-AP	1:2000
HO-1	Proteintech	10701-1-AP	1:2000
MRP2	NOVUS	NBP1-69023	1:1000
MRP3	Cell Signaling Technology	39909	1:1000
MRP4	Cell Signaling Technology	12857	1:1000
NQO1	Proteintech	11451-1-AP	1:2000
NRF2	Proteintech	16396-1-AP	1:1000
SOD2	Proteintech	24127-1-AP	1:5000
Tubulin	Proteintech	11224-1-AP	1:5000
β-actin	Proteintech	20536-1-AP	1:5000
