# Supplementary material for: Activation of Nrf2/HO-1 signaling pathway exacerbates cholestatic liver injury
Source: Commun Biol. 2024 May 23;7:621. doi: 10.1038/s42003-024-06243-0 (PMC11116386; doi:10.1038/s42003-024-06243-0)
Supplement: Supplementary file 3 — Description of Additional Supplementary Files [file 42003_2024_6243_MOESM3_ESM.pdf]

Description of Additional Supplementary Files

File name: Supplementary Data 1

Description: The numerical source data underlying graphs.

File name: Supplementary Data 2

Description: The processed sequencing data underlying Figure 7.

File name: Supplementary Data 3

Description: The source data behind human samples (include the source data for Figure 7, Supplementary Table 1 and patient information).
